# Identification of Common Differentially Expressed Genes and Potential Therapeutic Targets in Ulcerative Colitis and Rheumatoid Arthritis

**DOI:** 10.3389/fgene.2020.572194

**Published:** 2020-11-11

**Authors:** Yueying Chen, Hanyang Li, Lijie Lai, Qi Feng, Jun Shen

**Affiliations:** ^1^Division of Gastroenterology and Hepatology, Key Laboratory of Gastroenterology and Hepatology, Ministry of Health, Inflammatory Bowel Disease Research Center, Renji Hospital, School of Medicine, Shanghai Jiao Tong University, Shanghai Institute of Digestive Disease, Shanghai, China; ^2^Department of Radiology, Renji Hospital, School of Medicine, Shanghai Jiao Tong University, Shanghai, China

**Keywords:** bioinformatical analysis, hub genes, ulcerative colitis, rheumatoid arthritis, differentially expressed genes

## Abstract

Ulcerative colitis (UC) and rheumatoid arthritis (RA) are immune-mediated inflammatory diseases (IMIDs) with similar symptoms and common genomics. However, the relationship between UC and RA has not been investigated thoroughly. Therefore, this study aimed to establish the differentially expressed genes (DEGs) and potential therapeutic targets in UC and RA. Three microarray datasets (GSE38713, GSE1919, and GSE12251) were selected from the Gene Expression Omnibus (GEO) database for analysis. We used R software to identify the DEGs and performed enrichment analyses. Search Tool for the Retrieval of Interacting Genes/Proteins (STRING) and Cytoscape software were used to construct the protein-protein interaction (PPI) network and identify the hub genes. A regulatory network based on the constructed PPI was generated using StarBase and PROMO databases. We identified a total of 1542 and 261 DEGs in UC and RA. There were 169 common DEGs identified in both UC and RA, including 63 upregulated genes (DEGs1) and nine downregulated genes (DEGs2). The Gene Ontology (GO) and Kyoto Encyclopedia of Genes and Genomes (KEGG) pathway analyses of DEGs1 and DEGs2 in the PPI network revealed that the genes enriched were involved in immunity. A total of 45 hub genes were selected based on high scores of correlation; three hub genes (SRGN, PLEK, and FCGR3B) were found to be upregulated in UC and RA, and downregulated in UC patients with response to infliximab treatment. The identification of novel DEGs and hub genes in the current study contributes to a novel perception for latent functional mechanisms and presents potential prognostic indicators and therapeutic targets in UC and RA.

## Introduction

Ulcerative colitis (UC) is a chronic inflammatory disease that mainly involves the colon. The incidence and prevalence of UC have increased worldwide, thus placing a significant burden on human society (Ng et al., 2018). The intestinal symptoms that accompany UC include bloody diarrhea, and a third of patients with UC present with extraintestinal manifestations. Among these manifestations, arthritis has been the most commonly identified (Ungaro et al., 2017). Rheumatoid arthritis (RA) is an autoimmune disease that is characterized by inflammation, stiffness of joints accompanied by pain, loss of mobility, and joint deformity, and its incidence has increased substantially in the past 30 years (Safiri et al., 2019).

Studies report that patients with UC have an increased risk of RA (Wilson et al., 2016; Bae et al., 2017; Halling et al., 2017). UC and RA are immune-mediated inflammatory diseases (IMIDs); hence, they likely share similar pathogenesis, genes, and antigens. Previous studies have revealed several common genes associated with both UC and RA, including the human leukocyte antigen (*HLA-B27*), interleukin 15, peptidyl arginine deiminase type 4 (*PADI4*), and prostaglandin receptor EP4 (*PTGER4*) (Klausen et al., 1992; Mosquera-Martinez, 2001; Chen et al., 2008; Perdigones et al., 2010). UC and RA also share some common drugs for their treatment. TNF-α antagonists such as infliximab, have been approved as first- or second-line treatment of patients with UC and RA (Rubin et al., 2019; Smolen et al., 2020).

Despite extensive research on UC and RA, there still is a gap in understanding differentially expressed genes (DEGs) and possible targets for the treatment of UC and RA. Our study aimed to determine DEGs and possible targets for the treatment of UC and RA through bioinformatical analysis. In this study, we analyzed three gene expression datasets (GSE38713, GSE1919, and GSE12251) downloaded from the Restructured Gene Expression Omnibus (ReGEO) database. Comprehensive bioinformatics and enrichment analyses were used to determine independent DEGs and differentially coexpressed genes (DCGs). We constructed a protein-protein interaction (PPI) network to identify hub genes using the Search Tool for the Retrieval of Interacting Genes/Proteins (STRING) database and Cytoscape ver. 3.7.2 software. Moreover, we identified four potential therapeutic target genes related to UC and RA and constructed their regulatory network, using starBase and PROMO databases. These target genes include those of microRNAs (miRNAs), long non-coding RNAs (lncRNAs), and transcription factors (TFs). The potential therapeutic targets between UC and RA identified here are expected to provide novel insights into the biological mechanisms linked with these two diseases.

## Materials and Methods

### Data Source

GEO^1^ is a public repository containing high throughout sequencing and microarray data sets. We selected three gene expression microarray datasets (GSE38713, GSE1919, and GSE12251) from the GEO database. The GSE38713 and GSE12251 datasets were available on the GPL570 platform (HG-U133_Plus_2; Affymetrix Human Genome U133 Plus 2.0 Array), while GSE1919 was accessible on the GPL91 platform (HG_U95A; Affymetrix Human Genome U95A Array).

### Identification of DEGs

The R software (version 3.6.3)^2^ and limma package^3^ in Bioconductor^4^ were used to detect the DEGs affected by UC, RA, infliximab treatment response samples, and corresponding control groups (Ritchie et al., 2015). DEGs were identified using the selection criteria of adjusted *P*-value < 0.05 and | logFC| >1.0. The intersecting parts of DEGs were calculated using a Venn diagram webtool^5^.

### Gene Ontology and Pathway Enrichment Analysis of DEGs

Gene Ontology (GO) is a universal tool for defining the biological process (BP), cellular component (CC), and molecular function (MF) of numerous genes. Kyoto Encyclopedia of Genes and Genomes (KEGG) pathway is a database that contains multiple biological pathways for several organisms. GO and pathway analysis provide a deep insight into the relations of functions or pathways, and the primary roles of these genes. The enrichment analyses of DEGs were performed using the Cluster Profile package^6^ in Bioconductor, and a *P*-value less than 0.05 was considered as statistically significant (Yu et al., 2012).

### Protein-Protein Interaction Network Construction and Module Analysis

Protein-protein interaction (PPI) network reveals the specific and unspecific interactions of proteins, and identifies the core protein genes. STRING (version 11.0)^7^, is a freely accessible database, that collects, scores, and integrates data, is used to predict functional relationships between proteins (Szklarczyk et al., 2019). A PPI network of the DEGs with combined score >0.4 in STRING was considered as a functional link, and was constructed using the Cytoscape software (version 3.7.2)^8^ (Smoot et al., 2011; Wang et al., 2016a, 2018; Li et al., 2017; Zhao et al., 2019). Subsequently, we used the MCODE plugin to identify densely connected modules from the PPI network with the criteria of K-core = 2, degree cutoff = 2, max depth = 100, and node score cutoff = 0.2 (Bandettini et al., 2012).

### Selection and Analysis of Hub Genes

The degree of protein nodes was calculated by using the Cytoscape plugin, CytoHubba, to find hub genes (Chin et al., 2014). In this study, hub genes were selected with degrees ≥10. Subsequently, we used the corrplot package in R software to calculate the correlation between hub genes based on Pearson correlation analysis.

### Construction of Regulatory Network

The network of genes and their corresponding miRNAs and lncRNAs was constructed using StarBase^9^, a publicly available database that mainly focuses on miRNA-target interactions (Li et al., 2014). The transcription factors (TFs) of genes were downloaded from PROMO^10^, a public database for predicting the TFs of various genes through DNA sequences (Farre et al., 2003). The above-mentioned tools were combined to construct a multi-factor regulation network.

## Results

### Identification of DEGs

**FIGURE 1:**
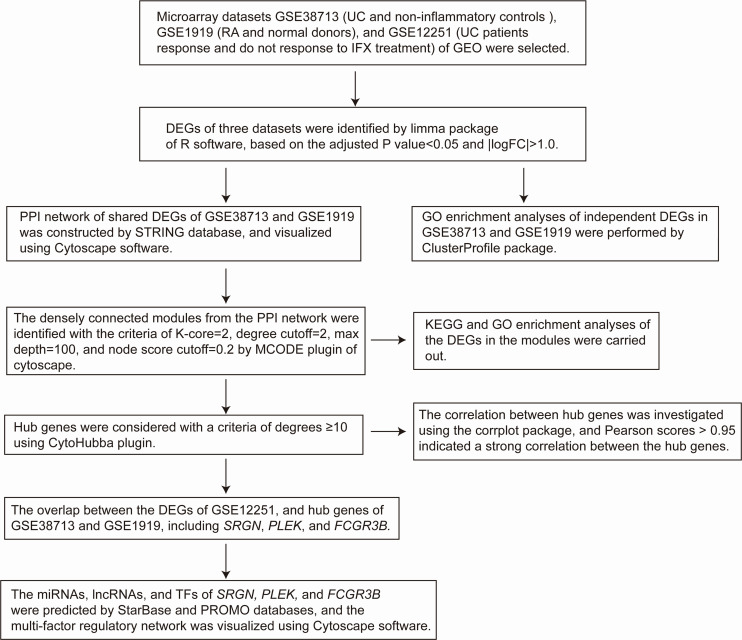
Flow diagram of the study design.

**FIGURE 2:**
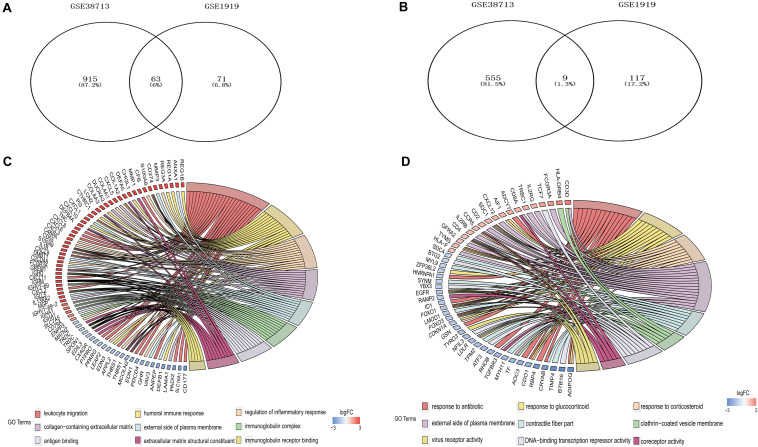
Differentially expressed genes (DEGs) among UC and RA. 63 upregulated DEGs **(A)** and 9 downregulated DEGs **(B)** expressed both in UC and RA. GO analyses of DEGs independently in UC **(C)** and RA **(D)** with adujust *P*-value.

Three gene expression datasets (GSE38713, GSE1919, and GSE12251) were selected in this study (Figure 1). The GSE38713 dataset was derived from 15 UC tissue samples and 10 control samples. GSE1919 dataset was derived from 5 RA samples and 5 control samples. GSE12251 dataset was derived from 22 patients with UC, of which 12 responded to and 10 did not respond to infliximab treatment. We used limma package to identify the DEGs in the three datasets with *P* < 0.05 and | logFC| >1. There were 1542 DEGs in the GSE38713 dataset, including 978 upregulated genes and 564 downregulated genes (Supplementary Table 1). There were 260 DEGs in the GSE1919 dataset, including 134 upregulated and 126 downregulated genes (Supplementary Table 2). In the GSE12251 dataset, 68 downregulated genes were identified (Supplementary Table 3). A Venn diagram was generated to show the overlap between GSE38713 and GSE1919 datasets; these include 63 upregulated genes (DEGs1) (Figure 2A) and 9 downregulated genes (DEGs2) (Figure 2B). Furthermore, 1470 DEGs (DEGs3) and 188 DEGs (DEGs4) were identified independently from the DEGs in UC and RA, respectively.

### GO Enrichment Analyses of Independent DEGs in UC and RA

GO analysis of GSE38713 indicated that the DEGs3 in UC were mainly involved in leukocyte migration, humoral immune response, and regulation of the inflammatory response under BP. The analysis also indicated that these DEGs were mainly involved in collagen-containing extracellular matrix, external side of plasma membrane, and immunoglobulin complex under CC. Likewise, the terms antigen binding, extracellular matrix structural constituent and immunoglobulin receptor binding were enriched under MF (Figure 2C). The GO analysis in the GSE1919 dataset for DEGs4 returned that the terms response to antibiotic, glucocorticoid, and corticosteroid under BP were mainly enriched. The terms enriched under CC were external side of plasma membrane, contractile fiber part, and clathrin-coated vesicle membrane. Moreover, the terms enriched under MF were DNA-binding transcription repressor activity, coreceptor activity, and virus receptor activity (Table 1 and Figure 2D).

**TABLE 1:** The GO enrichment analysis of DEGs3 and DEGs4 (top 3 terms according to *p*.adjust).

**DEGs**	**Ontology**	**ID**	**Description**	**Count**	***p*.adjust**
DEGs3	BP	GO:0050900	Leukocyte migration	138	3.44E-41
		GO:0006959	Humoral immune response	110	2.09E-37
		GO:0050727	Regulation of inflammatory response	120	1.06E-30
	CC	GO:0062023	Collagen-containing extracellular matrix	109	2.07E-32
		GO:0009897	External side of plasma membrane	98	1.92E-26
		GO:0019814	Immunoglobulin complex	53	3.78E-20
	MF	GO:0003823	Antigen binding	57	1.83E-21
		GO:0005201	Extracellular matrix structural constituent	47	1.61E-13
		GO:0034987	Immunoglobulin receptor binding	31	2.48E-13
DEGs4	BP	GO:0046677	Response to antibiotic	14	0.000109
		GO:0051384	Response to glucocorticoid	10	0.000109
		GO:0031960	Response to corticosteroid	10	0.000193
	CC	GO:0009897	External side of plasma membrane	16	1.88E-06
		GO:0044449	Contractile fiber part	8	0.008248
		GO:0030665	Clathrin-coated vesicle membrane	6	0.008248
	MF	GO:0001227	DNA-binding transcription repressor activity	8	0.000361
		GO:0015026	Coreceptor activity	5	0.005158
		GO:0001618	Virus receptor activity	5	0.020157

### Protein-Protein Interaction Network Construction and Module Analysis

**FIGURE 3:**
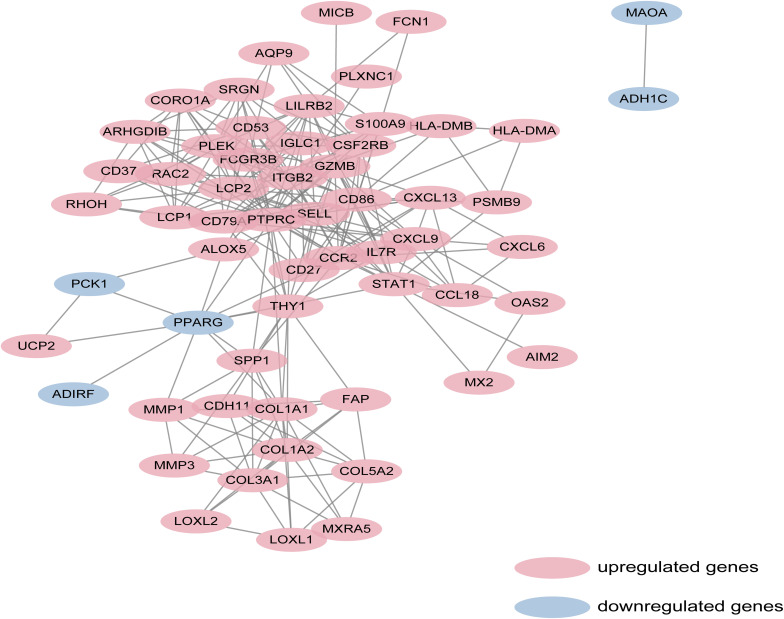
Based on database STRING and Cytoscape software, PPI networks of the DEGs1 and DEGs2 were constructed. The red point represents upregulated genes, and blue point represents downregulated genes.

**FIGURE 4:**
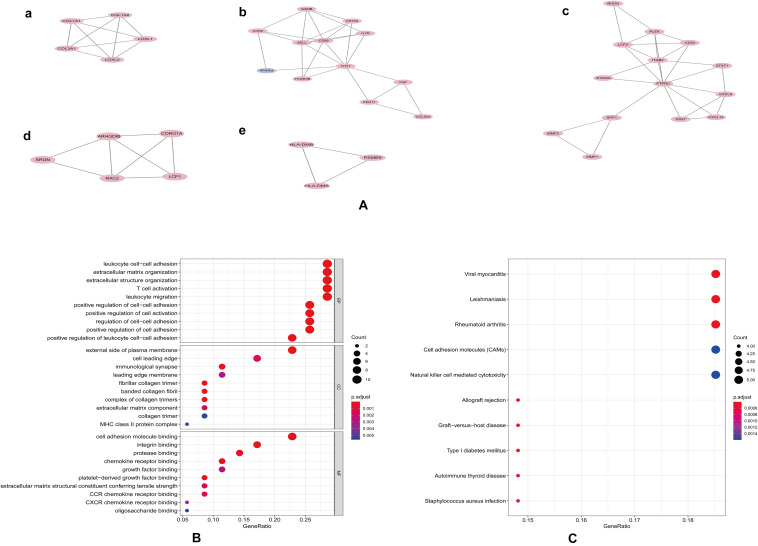
Modular analyses of PPI found five key modules **(A)**. GO **(B)** and KEGG **(C)** enrichment analyses of 39 genes in these modules with *P*-value.

Protein-protein interaction network analysis is a remarkable method in understanding the biological responses in health and disease. In this study, protein interactions between the DEGs1 and DEGs2 were analyzed using the STRING database. A total of 69 nodes and 251 edges were included with combined scores >0.4, and visualized using Cytoscape software (Figure 3). The MCODE plugin identified five densely connected modules in which 39 DEGs were among DEGs1 and DEGs2 (Figure 4A). KEGG and GO enrichment analyses of these 39 genes were carried out using the ClusterProfiler package. GO analysis revealed that these genes are involved in immunity (Figure 4B), and KEGG pathway analysis revealed them to be mainly involved in viral myocarditis, leishmaniasis, and allograft rejection (Figure 4C).

### Hub Gene Selection and Analysis

**FIGURE 5:**
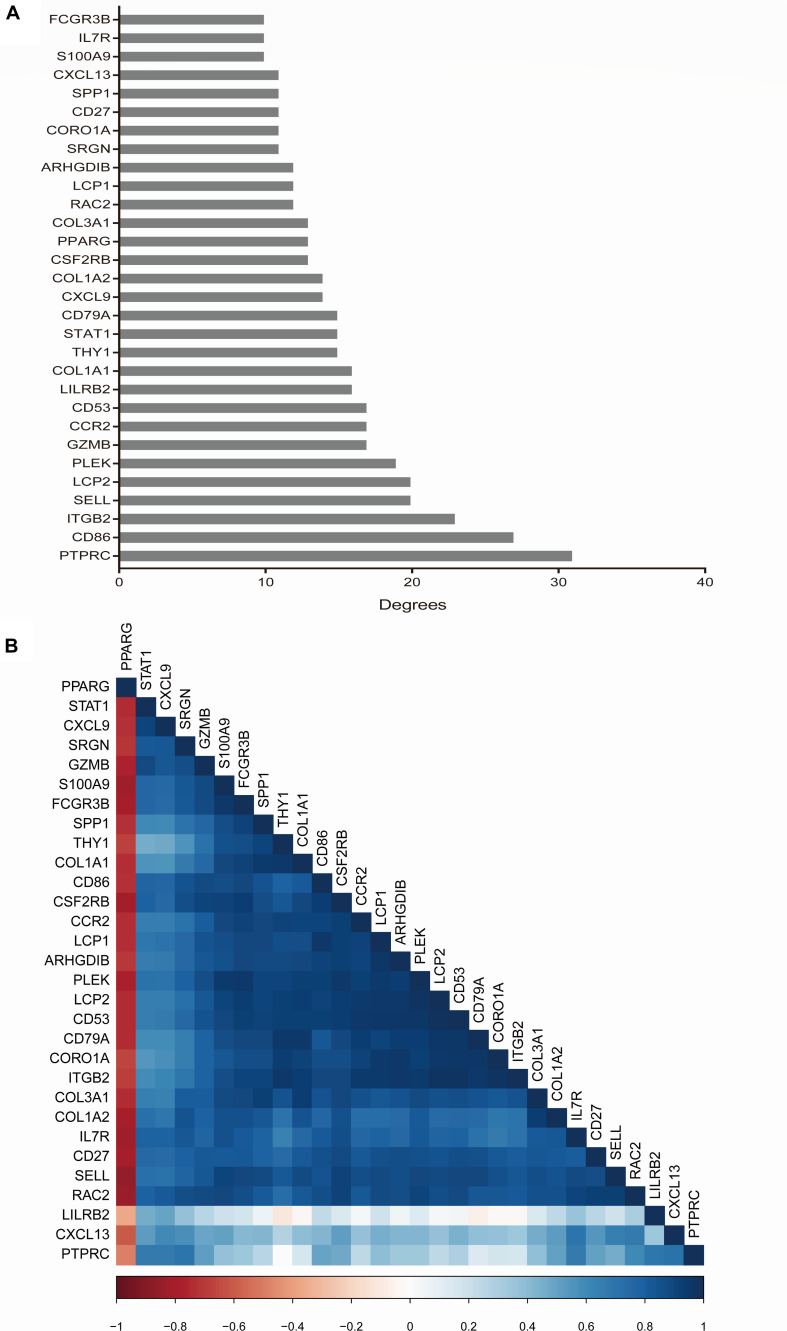
Hub gene selection and analysis performed by cytohubba. The score of hub genes was based on the EPC Algorithm **(A)**. Pearson correlation analysis was used to calculate the correlation of hub genes **(B)**.

With a criteria of degrees ≥10 using CytoHubba plugin, we identified a total of 30 hub genes. The scores of hub genes are presented in Figure 5A. The correlation between these 30 hub genes was investigated using the corrplot package, and Pearson scores >0.95 indicated a strong correlation between the hub genes. The correlation between 29 pairs of hub genes was considered significant (Figure 5B and Supplementary Table 4).

### Hub Genes in UC Response to Infliximab Treatment

We used limma packages to identify the DEGs in the GSE12251 dataset, and found 68 downregulated genes. The overlap between the hub genes of GSE38713, GSE1919, and GSE12251 include four protein-coding (pc) genes, such as *SRGN* (serglycin), *PLEK* (pleckstrin), and *FCGR3B* (Fc fragment of IgG receptor IIIb). All four genes were upregulated in UC and RA samples compared to those in the control samples in GSE38713 and GSE1919 datasets. On the other hand, these genes were downregulated in UC patients with response to infliximab treatment in the GSE12251 dataset. It could be concluded that the four genes play an important role during infliximab treatment of UC and RA.

### Multi-factor Regulation Network Construction

**FIGURE 6:**
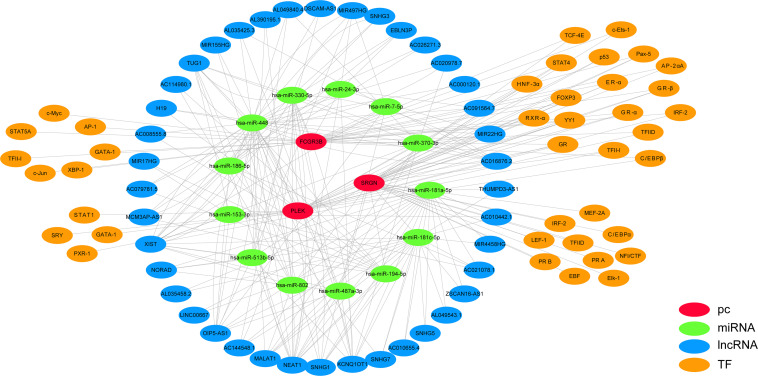
Multi-factor regulation network of *SRGN, PLEK*, and FCGR3B was constructed by starBase and PROMO database.

We used StarBase and PROMO databases to predict the miRNAs, lncRNAs, and TFs of *SRGN, PLEK*, and *FCGR3B*, and found a total of 16 miRNAs, 40 lncRNAs, and 41 TFs. The data of these four genes and their miRNAs, lncRNAs, and TFs were integrated into a regulatory network, and visualized using Cytoscape software (Figure 6).

## Discussion

There has been a considerable increase in the incidence and prevalence of UC and RA worldwide (Molodecky et al., 2012; Safiri et al., 2019). These diseases can lead to functional disabilities, severe decline in quality of life, and an increased risk of cancer (Jess et al., 2012; Simon et al., 2015; Myasoedova et al., 2019). Furthermore, UC has been reported to be concomitant with RA (Wilson et al., 2016). As IMIDs, UC and RA might have overlapping pathogenic pathways. Inflammatory and immune regulatory pathways, such as Fcγ receptor signaling, are linked to the pathogenesis of IMIDs (Castro-Dopico and Clatworthy, 2019; Virtanen et al., 2019). Additionally, gut microbiota has been reported to play a role in IMIDs (Liu et al., 2013; Forbes et al., 2018; Imhann et al., 2018). Treatment with TNF-α antagonists has been firmly established as an effective therapeutic approach for RA (Kievit et al., 2008); however, non-responsiveness to infliximab (a TNF-α antagonist) is common in patients with UC (Kievit et al., 2008; Wong and Cross, 2017). The main purpose of our study was to identify the common DEGs in UC and RA, thereby revealing potential targets for predicting the therapeutic effect of TNF-α antagonist and treating UC and RA.

In this study, we identified 72 overlapping DEGs in both UC and RA, of which 63 were upregulated (DEGs1) and 9 were downregulated genes (DEGs2). Independent DEGs included 915 upregulated and 555 downregulated genes in UC (DEGs3), and 71 upregulated and 117 downregulated genes in RA (DEGs4). GO analysis revealed that DEGs3 were significantly enriched in inflammatory and immune pathways, which played a central role in the development of UC and RA. While DEGs4 were mainly enriched in drug responses. Enrichment analyses of the genes in the key modules of the constructed PPI network revealed that they were mainly enriched in some immune-related pathways and cellular organization processing, such as leukocyte cell-cell adhesion, extracellular matrix organization, T cell activation, and leukocyte migration. The adhesion and migration of leukocyte, as well as the activation of T cells have been linked to the pathogenesis of UC and RA (Thomas and Baumgart, 2012; Reynisdottir et al., 2016; McNaughton et al., 2018; Rabe et al., 2019). Finally, a total of 45 hub genes were identified, among which four hub genes (*SRGN, PLEK*, and *FCGR3B*) were predicted to be upregulated in UC and RA samples (GSE38713 and GSE1919) and downregulated in UC patients with response to infliximab treatment (GSE12251). These findings suggest that these four genes are predictive markers and therapeutic targets for UC and RA.

*SRGN* encodes the proteoglycan protein, and is mainly expressed in hematopoietic cells. Many studies have confirmed that *SRGN* promotes tumor invasion and metastasis in colorectal cancer, non-small cell lung cancers, multiple myeloma, nasopharyngeal carcinoma, and breast cancer (Li et al., 2011; Korpetinou et al., 2013; Purushothaman and Toole, 2014; Guo et al., 2017; Xu et al., 2018). *SRGN* is also involved in inflammatory processes through the regulation of numerous inflammatory mediators such as TNF-α, and activating the NF-κB signaling pathway (Zernichow et al., 2006; Korpetinou et al., 2014; Scuruchi et al., 2019). These processes caused by the combination of *SRGN* and CD44 receptor, could promote inflammation (Misra et al., 2015). *PLEK*, a substrate of protein kinase C, is involved in various adaptive immune responses (Cremonesi et al., 2012). Although the underlying mechanisms of *PLEK* are still unclear, many studies have linked it to certain diseases. *PLEK* might be a susceptibility locus for venous thromboembolism, and its expression is increased in UC, periodontitis, and celiac disease (Song et al., 2015; Pascual et al., 2016; Lindstrom et al., 2019; Medrano et al., 2019). In diabetes, *PLEK* has been reported to promote the secretion of proinflammatory cytokines such as TNF-α and IL-1β in mononuclear phagocytes; these cytokines have already been linked to increased risk of UC and RA (Ding et al., 2007; Hermanns et al., 2016). We speculate that *SRGN* and *PLEK* are involved in the pathogenesis of UC and RA through the increase in inflammatory factors. Many researches have proven that the copy number variation (CNV) of *FCGR3B* is linked to autoimmune and inflammatory diseases. Low copy number and the deletion of *FCGR3B* increase the risk of RA (Tsang-A-Sjoe et al., 2016; Wang et al., 2016b; Rahbari et al., 2017; Zheng et al., 2017), and *FCGR3B* gene copy number has also been suggested to increase susceptibility to UC, which indicates that *FCGR3B* might be the key gene involved in their pathogenesis (Asano et al., 2013). Although the specific mechanisms of action of *Fc*γ*RIIIb* in IMIDs remain unclear, studies revealed that *Fc*γ*RIIIb* is a stimulatory Fc gamma receptor which promotes neutrophil recruitment and the capture and clearance of immune complexes (ICs); the deletion of FCGR3B lead to immune-complex-mediated diseases (Dijstelbloem et al., 2001; Willcocks et al., 2008; Chen et al., 2012). We speculate that *FCGR3B* is involved in the inflammatory processes in IgG-IC-FcγR signaling (Mathsson et al., 2006; Uo et al., 2013; Bersellini Farinotti et al., 2019).

The limitations of our study are as follows. Our study is a retrospective analysis and has small sample size. Hence, our findings need to be validated using a larger cohort and prospective studies. We did not assess the potential therapeutic roles of *SRGN, PLEK*, and *FCGR3B* in UC and RA; therefore, further clinical research is needed to investigate whether they could be used as predictive factors for infliximab efficacy in patients with UC and RA. Finally, we did not explore the specific mechanisms of these four genes in UC and RA, which warrants further studies.

## Conclusion

In conclusion, our study identified 169 novel DEGs and 45 hub genes common in both UC and RA. GO and KEGG analyses of independent DEGs and the hub genes in UC and RA might reveal a novel prospective relationship between UC and RA. In addition, we found four hub genes (*SRGN, PLEK*, and *FCGR3B)* that were significantly associated with infliximab treatment in UC. These genes need to be explored further for their clinical relevance as potential prognostic indicators and therapeutic targets in UC and RA.

## Data Availability Statement

The datasets presented in this study can be found in online repositories. The names of the repository/repositories and accession number(s) can be found in the article/Supplementary Material.

## Author Contributions

YC and HL collected the papers and analyzed data, analyzed the conclusions, and drafted the manuscript. LL reviewed the data and conclusions. JS presented the idea of this manuscript, supported the funding, analyzed the conclusions, drafted and revised the manuscript. All authors contributed to the article and approved the submitted version.

## Conflict of Interest

The authors declare that the research was conducted in the absence of any commercial or financial relationships that could be construed as a potential conflict of interest.

## Footnotes

1 http://www.ncbi.nlm.nih.gov/geo/

2 https://www.r-project.org/

3 http://www.bioconductor.org/packages/release/bioc/html/limma.html

4 http://www.bioconductor.org/

5 http://bioinfogp.cnb.csic.es/tools/venny/index.html

6 http://www.bioconductor.org/packages/release/bioc/html/clusterProfiler.html

7 http://string-db.org

8 http://www.cytoscape.org/

9 http://starbase.sysu.edu.cn/index.php

10 http://alggen.lsi.upc.es/cgi-bin/promo_v3/promo/promoinit.cgi?dirDB=TF_8.3

## Supplementary Material

Supplementary Table 1 Differentially expressed genes in GSE38713.

Supplementary Table 2 Differentially expressed genes in GSE1919.

Supplementary Table 3 Differentially expressed genes in GSE12251.

Supplementary Table 4 Correlations between hub genes.

## Abbreviations

BP: biological process
CC: cellular component
CNV: copy number variation
DEGs: differentially expressed genes
FCGR3B: Fc fragment of IgG receptor IIIb
ICs: immune complexes
lncRNAs: long non-coding RNAs
IMIDs: immune-mediated inflammatory diseases
MF: molecular function
miRNAs: microRNAs
NFkB: nuclear transcription factor kappaB
OS: overall survival
PPI: protein-protein interaction
*PLEK*: pleckstrin
RA: rheumatoid arthritis
*SRGN*: serglycin
TF: transcription factors
UC: ulcerative colitis.
