# Supplementary material for: Identification of Common Differentially Expressed Genes and Potential Therapeutic Targets in Ulcerative Colitis and Rheumatoid Arthritis
Source: Front Genet. 2020 Nov 11;11:572194. doi: 10.3389/fgene.2020.572194 (PMC7686785; doi:10.3389/fgene.2020.572194)
Supplement: Supplementary file 6 [file Table_6.DOCX]

**Table 1.** The GO enrichment analysis of DEGs3 and DEGs4 (top 3 terms according to p.adjust)

| DEGs | ontology | ID | description | Count | p.adjust |
| --- | --- | --- | --- | --- | --- |
| DEGs3 | BP | GO:0050900 | leukocyte migration | 138 | 3.44E-41 |
|  |  | GO:0006959 | humoral immune response | 110 | 2.09E-37 |
|  |  | GO:0050727 | regulation of inflammatory response | 120 | 1.06E-30 |
|  | CC | GO:0062023 | collagen-containing extracellular matrix | 109 | 2.07E-32 |
|  |  | GO:0009897 | external side of plasma membrane | 98 | 1.92E-26 |
|  |  | GO:0019814 | immunoglobulin complex | 53 | 3.78E-20 |
|  | MF | GO:0003823 | antigen binding | 57 | 1.83E-21 |
|  |  | GO:0005201 | extracellular matrix structural constituent | 47 | 1.61E-13 |
|  |  | GO:0034987 | immunoglobulin receptor binding | 31 | 2.48E-13 |
| DEGs4 | BP | GO:0046677 | response to antibiotic | 14 | 0.000109 |
|  |  | GO:0051384 | response to glucocorticoid | 10 | 0.000109 |
|  |  | GO:0031960 | response to corticosteroid | 10 | 0.000193 |
|  | CC | GO:0009897 | external side of plasma membrane | 16 | 1.88E-06 |
|  |  | GO:0044449 | contractile fiber part | 8 | 0.008248 |
|  |  | GO:0030665 | clathrin-coated vesicle membrane | 6 | 0.008248 |
|  | MF | GO:0001227 | DNA-binding transcription repressor activity | 8 | 0.000361 |
|  |  | GO:0015026 | coreceptor activity | 5 | 0.005158 |
|  |  | GO:0001618 | virus receptor activity | 5 | 0.020157 |
