# Supplementary material for: Identification of Common Differentially Expressed Genes and Potential Therapeutic Targets in Ulcerative Colitis and Rheumatoid Arthritis
Source: Front Genet. 2020 Nov 11;11:572194. doi: 10.3389/fgene.2020.572194 (PMC7686785; doi:10.3389/fgene.2020.572194)
Supplement: Supplementary file 5 [file Table_5.DOCX]

Jun13th, 2020

Dear Editor:

I wish to submit an original article for publication in *Frontiers in Genetics*, titled “**Identification of common differentially expressed genes and potential therapeutic targets in ulcerative colitis and rheumatoid arthritis**.” The paper was coauthored by Chen Yueying, Li Hanyang, and Lai Lijie.

Ulcerative colitis (UC) and rheumatoid arthritis (RA) are immune-mediated inflammatory diseases (IMIDs) that are reported to share similar pathogenesis, genes, and antigens. In spite of intense research in the field of UC and RA, there is still a major gap in our understanding of the differentially expressed genes (DEGs) and possible targets in the treatment of UC and RA. Therefore, this study aimed to establish the DEGs and potential therapeutic targets in UC and RA. We analyzed three gene expression datasets downloaded from the Gene Expression Omnibus (GEO) database. Comprehensive bioinformatics analysis was used to detect the independent and co-expressive DEGs as well as the enrichment analysis of DEGs. We identified four potential therapeutic target genes related to UC and RA and constructed their multi-factor regulation network using starBase and PROMO databases. We identified 72 overlapping DEGs in both UC and RA, which contained 63 up-regulated and 9 down-regulated genes. Independent DEGs contained 915 up-regulated and 555 down-regulated genes in UC, and 71 up-regulated and 117 down-regulated genes in RA. Enrichment analyses revealed that the genes were mainly enriched in some immune-related pathways and cellular organization processing, such as leukocyte cell-cell adhesion, extracellular matrix organization, T cell activation, and leukocyte migration. Finally, a total of 45 hub genes were identified among which four hub genes (*AQP9, SRGN, PLEK,* and *FCGR3B*) were found to be up-regulated in UC and RA samples, while the expressions of these hub genes were decreased in case of response to infliximab treatment in patients with UC. These findings suggest that these four genes might be potential predictive markers and therapeutic targets for UC and RA.

As your esteemed journal publishes articles covering all the biological contexts of genes, we believe that the findings of this study align with the aims and scope of the journal, and will be of interest to its readership.

Please consider, as potential referees, Kouichi Asano, Kyushu University, k-asano@intmed2.med.kyuishu-u.ac.jp; Kazuhiro Tsukamoto, Nagasaki University Graduate School of Biomedical Sciences, ktsuka@nagasaki-u.ac.jp; Kim KJ, The Catholic University of Korea, md21c@catholic.ac.kr; Jianping Wang, Sun Yat-sen University, wjp@mail.sysu.edu.cn.

This manuscript has not been published or presented elsewhere in part or in entirety and is not under consideration by another journal. The need for informed consent was waived off as this was a retrospective study. We have read and understood your journal’s policies, and we believe that neither the manuscript nor the study violates any of these. There are no conflicts of interest to declare.

Thank you for your consideration. I look forward to hearing from you.

Sincerely,

Jun Shen, M.D. and Ph.D.

Associate Professor

145 Shan Dong Zhong Road

Department of Gastroenterology

Renji Hospital, Shanghai Jiao Tong University, School of Medicine

Shanghai Institute of Digestive Disease, Shanghai 200001, P. R. China

E-mail: shenjun_renji@163.com

Tel: +86 21 63260930

Fax: + 86 21 63266027
